# A_2A_ adenosine receptor-driven cAMP signaling in olfactory bulb astrocytes is unaffected in experimental autoimmune encephalomyelitis

**DOI:** 10.3389/fimmu.2023.1273837

**Published:** 2023-11-23

**Authors:** Marina Wendlandt, Alina J. Kürten, Antonia Beiersdorfer, Charlotte Schubert, Kiana Samad-Yazdtchi, Jessica Sauer, M. Carolina Pinto, Kristina Schulz, Manuel A. Friese, Christine E. Gee, Daniela Hirnet, Christian Lohr

**Affiliations:** ^1^ Division of Neurophysiology, University of Hamburg, Hamburg, Germany; ^2^ Institute of Neuroimmunology and Multiple Sclerosis (INIMS), University Medical Center Hamburg-Eppendorf, Hamburg, Germany; ^3^ Institute of Synaptic Physiology, Center for Molecular Neurobiology Hamburg, Hamburg, Germany

**Keywords:** cyclic adenosine monophosphate, astrocyte, purinergic signaling, adenosine, olfactory bulb, cAMP imaging, experimental autoimmune encephalomyelitis, neuroinflammation

## Abstract

**Introduction:**

The cyclic nucleotide cyclic adenosine monophosphate (cAMP) is a ubiquitous second messenger, which is known to play an important anti-inflammatory role. Astrocytes in the central nervous system (CNS) can modulate inflammation but little is known about the significance of cAMP in their function.

**Methods:**

We investigated cAMP dynamics in mouse olfactory bulb astrocytes in brain slices prepared from healthy and experimental autoimmune encephalomyelitis (EAE) mice.

**Results:**

The purinergic receptor ligands adenosine and adenosine triphosphate (ATP) both induced transient increases in cAMP in astrocytes expressing the genetically encoded cAMP sensor Flamindo2. The A_2A_ receptor antagonist ZM241385 inhibited the responses. Similar transient increases in astrocytic cAMP occurred when olfactory receptor neurons were stimulated electrically, resulting in ATP release from the stimulated axons that increased cAMP, again via A_2A_ receptors. Notably, A_2A_-mediated responses to ATP and adenosine were not different in EAE mice as compared to healthy mice.

**Discussion:**

Our results indicate that ATP, synaptically released by afferent axons in the olfactory bulb, is degraded to adenosine that acts on A_2A_ receptors in astrocytes, thereby increasing the cytosolic cAMP concentration. However, this pathway is not altered in the olfactory bulb of EAE mice.

## 1 Introduction

Astrocytes are the most abundant glial cells in the mammalian central nervous system. They fulfill a variety of functions in development, homeostasis and physiology of the brain, including extracellular K^+^ and neurotransmitter clearance, metabolic support of neurons, neurovascular coupling and synaptic plasticity (1, 2). In addition to the multifaceted functions of astrocytes in brain homeostasis and performance, it becomes increasingly clear that astrocytes play a pivotal role in the initiation and progression of mental and neurological disorders such as depression, Alzheimer’s disease, stroke and epilepsy (3–7). In a neuroinflammatory environment as found in multiple sclerosis (MS), astrogliosis has been described, leading to astrocyte activation, hypertrophy, release of cytokines and increased glutamate release (6, 8–12). Reactive astrogliosis is affected by purinergic signaling and, in turn, modulates purinergic signaling in other cell types (13). For example, in reactive astrocytes in mouse models of epilepsy and stroke, P2Y_1_ receptor-mediated Ca^2+^ signaling is elevated (14, 15) resulting in increased release of cytokines and chemokines (16, 17).

Most astrocyte functions are triggered or modulated by Ca^2+^ signaling (2, 18). Much less is known about the equally important and ubiquitous second messenger, cyclic adenosine monophosphate (cAMP) (19). cAMP is generated by adenylyl cyclases, a family of intracellular enzymes which may be stimulated by one group of G proteins (G_s_ proteins) and inhibited by another group of G proteins (G_i_ proteins) (20). Hence, the concentration of cAMP in cells may be controlled by a combination of G_s_- and G_i_-coupled receptors. Downstream of cAMP production are cAMP-dependent proteins such as cAMP-dependent protein kinase A (PKA), exchange protein activated by cAMP (EPAC), and two types of ion channels, namely cyclic nucleotide-gated (CNG) and hyperpolarization-activated cyclic nucleotide-gated (HCN) ion channels (21). In astrocytes, several neurotransmitter receptors linked either to G_s_ or G_i_ have been described, however, only few studies visualized cAMP dynamics in this cell type due to the relatively recent development of suitable single-wavelength genetically encoded cAMP indicators and the lack of chemical cAMP indicators (22–24).

In the present study, we aimed to visualize cAMP signals in astrocytes in the olfactory bulb, one of the brain regions that is first affected in neurodegenerative disorders (25, 26). We expressed the genetically encoded cAMP indicator Flamindo2 (27) in astrocytes and imaged cAMP in acute brain slices of olfactory bulbs from healthy mice and mice suffering from experimental autoimmune encephalomyelitis (EAE), an animal model for multiple sclerosis (MS). Our results show that neuronal release of adenosine-triphosphate (ATP) and degradation to adenosine results in transient increases of the intracellular cAMP concentration mediated by A_2A_ adenosine receptors in olfactory bulb astrocytes. Increases in cAMP were significantly larger in somata compared to cell processes. No difference between astrocyte cAMP signals in healthy control mice and mice with EAE could be detected. Our results indicate that A_2A_ receptors in olfactory bulb astrocytes trigger cAMP signals that are not affected by neuroinflammation.

## 2 Materials and methods

### 2.1 Animals

All imaging experiments were performed on 6–16 weeks old C57Bl/6J wild type mice of both sexes (for EAE see below) (The Jackson Laboratory, Bar Harbor, ME, USA). Mice were held in the animal facilities of the Institute of Cell and Systems Biology (University of Hamburg) and the University Medical Center Hamburg-Eppendorf (UKE). Mice were kept in a 12/12 light/dark cycle and had access to food and water *ad libitum*. All experiments were performed in accordance with German law, the directive 2010/63/EU and were approved by the local ethics committee and the Behörde für Justiz und Verbraucherschutz, Lebensmittel und Veterinärwesen Hamburg.

### 2.2 Virus injection, Flamindo2 and GRAB_ATP1.0_ expression

To express the genetically encoded cAMP indicator Flamindo2 specifically in astrocytes, we used endotoxin-free recombinant adeno-associated viruses AAV^2/PhP.eB^hGFAP-Flamindo2 and AAV^2/PhP.AX^ hGFAP-Flamindo2 that were prepared at the UKE vector facility. The plasmid pAAV-hGFAP-Flamindo2 was a gift provided by Hajime Hirase, pAAV-gfaABC1D-GRAB_ATP1.0 Addgene #167579 was a gift from Yulong Li, the capsid plasmid PhP-AX Addgene #195218 was kindly provided by Vivianna Gradinaru and Xinhong Chen and the capsid plasmid PhP.eB Addgene #103005 was a gift from V. Gradinaru (27–30). Virus suspensions were diluted with sterile saline and injected i.v. into the orbital sinus of isoflurane-anesthetized mice. Each animal received a total volume of 70 µl virus suspension containing 1 x 10^11^ viral genomes (quantified by qPCR for WPRE sequence).

### 2.3 Solutions

Experiments were performed in artificial cerebrospinal fluid (ACSF) that contained (in mM): NaCl, 120; NaHCO_3_, 26; NaH_2_PO_4_, 1; KCl, 2.5; D-glucose, 2.8; CaCl_2_, 2; MgCl_2_, 1. For preparation of brain slices, Na^+^- and Ca^2+^-reduced ACSF was used (in mM): NaCl, 83; NaHCO_3_, 26.2; NaH_2_PO_4_, 1; KCl, 2.5; CaCl_2_, 0.5; MgSO_4_, 2.5. In Na^+^- and Ca^2+^-reduced ACSF, D-glucose was increased to 20 mM and sucrose was added (70 mM) to compensate for reduced Na^+^ concentration and maintain osmolarity. The purinergic ligands ATP and adenosine were purchased from Merck/Sigma-Aldrich (Taufkirchen, Germany), N-Cyclopentyladenosine (N^6^-CPA), 2-Chloro-N-cyclopentyl-2’-methyladenosine (MeCCPA), 4-[2-[(6-Amino-9-b-D-ribofuranosyl-9H-purin-2-yl)thio]ethyl]benzenesulfonic acid ammonium salt (PSB 0777) from Bio-techne (Wiesbaden, Germany), forskolin and 4-(2-[7-Amino-2-(2-furyl) [1,2,4]triazolo [2,3-a][1,3,5]triazin-5-ylamino]ethyl) phenol (ZM241385) from Abcam (Cambridge, UK) and tetrodotoxin (TTX) from Biozol (Hamburg, Germany). Stock solutions were prepared according to the manufacturer’s instructions, dissolved in ACSF at the final concentrations given in the results section and applied with the perfusion system.

### 2.4 Preparation of brain slices

Mice were anesthetized using isoflurane (5% v/v in oxygen) and decapitated. Brains were quickly dissected and transferred into chilled Ca^2+^-reduced ACSF that was continuously gassed with carbogen (95% O_2_/5% CO_2_; buffered to pH 7.4 with CO_2_/bicarbonate) during the entire preparation procedure. Two hundred micrometer thick slices of the bulbs were cut using a vibratome (VT1200S, Leica). Slices were stored in ACSF gassed with carbogen in darkness for 30 min at 30°C and then at room temperature until starting experiments.

### 2.5 Confocal cAMP imaging and electrical stimulation

Changes in the intracellular cAMP concentration were visualized by the genetically encoded cAMP indicator Flamindo2, based on the circular permuted fluorescent protein citrine and the cAMP binding domain of mEPAC1 (27). Binding of cAMP to the cAMP binding site decreases the fluorescence intensity of citrine, hence an increase in cAMP concentration is reflected by a decrease in fluorescence intensity. Flamindo2 was excited at 488 nm and fluorescence was collected between 500 and 530 nm using a confocal microscope (eC1, Nikon). Time series of images were recorded at a frame rate of 1 frame every 3-5 seconds. To visualize increases in the extracellular ATP concentration, we expressed the EGFP-based fluorescent ATP sensor GRAB_ATP1.0_ in astrocytes, which was excited at 488 nm and emitted light in the range of 500-530 nm (30). Increases in extracellular ATP were evoked by electrical stimulation of axons of olfactory receptor neurons that release ATP at their axon terminals. To stimulate these axons and trigger action potentials, a glass micropipette (GC150TF-10, Harvard Apparatus, USA) pulled with a patch-pipette puller (Flaming/Brown P97, Sutter Instruments, Novato, CA, USA) with electrical resistance of 1-3 MΩ was inserted into the olfactory nerve layer of a brain slice and trains of electrical pulses (0.1 ms pulse width, 2.3 mA amplitude, 20 Hz for 10 s) were applied (Digitimer DS3, Digitimer Ltd, Hertfordshire, England).

### 2.6 Immunohistochemistry

Successful infection and astrocyte-specific expression of Flamindo2 was verified by co-immunolabeling of Flamindo2 and astrocytic markers. Since Flamindo2 is based on citrine, a yellow-fluorescent variant of GFP, we used anti-GFP (chicken, #132 006, Synaptic Systems, Göttingen, Germany) to visualize Flamindo2 and a combination of anti-GFAP (rabbit, Z0334, Dako, Hamburg, Germany) and anti-S100 (rabbit, Z0311, Dako) to visualize astrocytes, all primary antibodies were used at a concentration of 1:500. Free floating slices (220 µm thick) were cut from brains fixed with 4% formalin using a vibratome (VT1000, Leica, Bensheim, Germany). Slices were rinsed with PBS (in mM: NaCl, 130; Na_2_HPO_4_, 7; NaH_2_PO_4_, 3) and blocked with 10% normal goat serum (NGS) and 0.5% Triton X-100 in PBS for one hour. Primary antibodies were incubated in 1.0% NGS and 0.05% Triton X-100 in PBS at 4°C for 36 hours. Secondary antibodies (Alexa Fluor 488 goat anti-chicken, #Ab150173, Abcam, Cambridge, UK; Alexa Fluor 555 goat anti-rabbit, #A21429, Life Technologies GmbH, Darmstadt, Germany) were incubated in PBS at 4°C overnight. After rinsing in PBS slices were mounted in Shandon Immu-Mount (Life Technologies GmbH) and recorded using a confocal microscope (eC1, Nikon, Düsseldorf, Germany). Since both Flamindo2 and Alexa Fluor 488 emission was recorded in the same range of wavelengths (500-530 nm), both signals add up to optimize the visualization of Flamindo2-expressing cells.

### 2.7 Experimental autoimmune encephalomyelitis

Mice were acclimatized two weeks before EAE induction in the experimental housing facility. EAE induction was performed as described before (31). Briefly, 13 to 14-week-old female C57Bl/6J mice were anesthetized with isoflurane (1% to 2% v/v in oxygen). Immunization was performed by a subcutaneous injection into the two lateral sides of the lower back area. Injections consisted of 200 µg myelin oligodendrocyte glycoprotein peptide (MOG35–55, peptides & elephants GmbH, Hennigsdorf, Germany) in saline mixed with complete Freund’s adjuvant (CFA, BD Difco) containing 4 mg/ml heat-killed Mycobacterium tuberculosis (BD Difco). On the day of immunization and two days later, 250 ng pertussis toxin (Merck Millipore) in 100 µl PBS was injected intraperitoneally. Control mice were not treated. Animals were weighed and scored daily for the presence of neurological signs from day 7 to the day of preparation. Mice were scored for clinical signs by the following system: 0: no clinical deficits; 1: tail weakness; 2: hind limb paresis; 3: partial hind limb paralysis; 3.5: full hind limb paralysis; 4: full hind and forelimb paralysis or plegia. Animals that reached a score of 4, or scored 3.5 for more than 7 days, or lost ≥ 25% of starting weight were euthanized according to the regulations of the local Animal Welfare Act. At the peak of the disease, the mean EAE score was 2.8 ± 1.16.

### 2.8 Data analysis and statistics

Data were evaluated with Nikon EZ-C1 Viewer (Nikon), processed using Excel (Microsoft, USA) and statistical tests were applied with Origin Pro 9.1 (OriginLab Corporation, Northampton, USA). The expression of Flamindo2 was highest in astrocytes in the glomerular layer. We therefore only analyzed astrocytes in the glomerular layer. Somata of Flamindo2-expressing astrocytes were marked by a region of interest (ROI) and the mean fluorescence intensity within the ROI was measured throughout the time series. In a subset of experiments, cell processes were analyzed in addition to somata. The mean fluorescence intensity (F) was normalized to the basal fluorescence intensity at rest which was set to 100%, hence changes in fluorescence intensity are stated as ΔF in %. Since an increase in the cAMP concentration in astrocytes is reported as a decrease in Flamindo2 fluorescence, we inverted the fluorescence traces in the figures to render perception of the results more intuitively.

Fluorescence changes of less than 5% ΔF were in the range of noise and were not counted as responses. All data values are given as mean ± standard error of the mean (SEM) with n representing the number of astrocytes analyzed. For each set of experiments, at least three animals were used. Means were tested for statistical differences using the Mann-Whitney-U test. For multiple comparisons the Kruskal-Wallis ANOVA followed by Dunn’s *post hoc* test was used. Statistical differences were indicated with *p < 0.05, **p < 0.01 and ***p < 0.001. Bar graphs and the sigmoidal fit of the dose-response curve were prepared using OriginPro 9.1. Bar graphs represent means + SEM. They were overlaid by the entire population of single data points that were normalized to the mean of the first application/stimulation.

## 3 Results

### 3.1 Adenosine increases the cAMP concentration in olfactory bulb astrocytes

**Figure 1:**
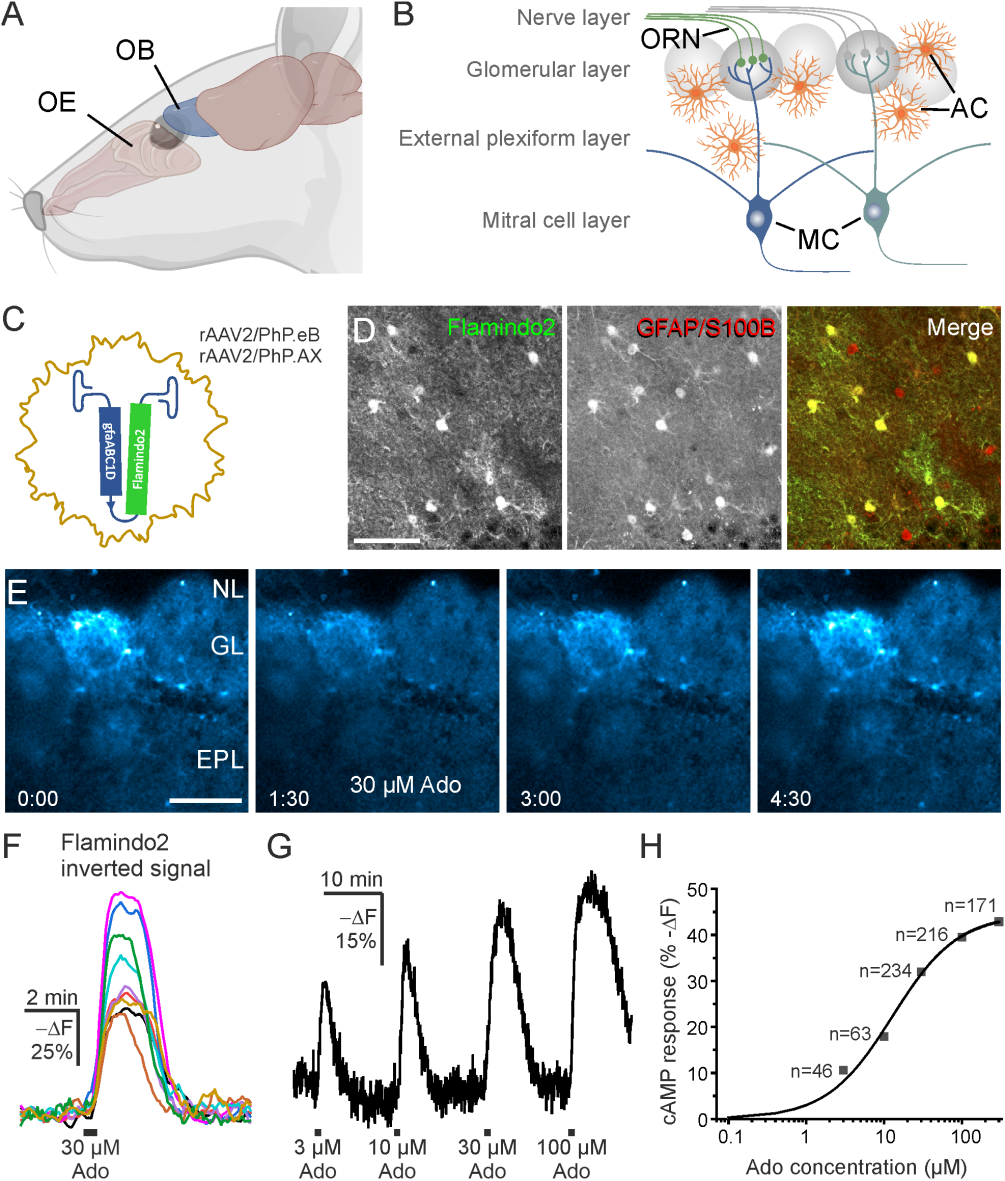
Adenosine increases cAMP in olfactory bulb astrocytes. **(A)** Location of olfactory bulb (OB) relative to the nose and olfactory epithelium (OE). **(B)** Organization of the OB. Input axons from olfactory receptor neurons (ORN) in the olfactory epithelium form synapses onto neurites of the mitral cells (MC) in the glomerular layer. AC, astrocytes. **(C)** Recombinant adeno associated viral vectors rAAV^2/PhP.eB^gfaABC1D-Flamindo2 or rAAV^2/PhP.AX^gfaABC1D-Flamindo2 were used to transduce OB astrocytes with the cAMP indicator Flamindo2 following i.v. injection. **(D)** Antibody staining verified that Flamindo2 (anti-GFP, left panel) was expressed in astrocytes (anti-GFAP/anti-S100B, middle panel). The right panel depicts merged images of Flamindo2 (green) and astrocyte (red) channels. Note absence of green-only astrocytes. Scale bar 50 µM. **(E)** Image series of Flamindo2 fluorescence immediately before (0:00 min:s), during (1:30 min:s) and at later time points after 30 s bath application of 30 µM adenosine (Ado). Scale bar 100 µM. **(F)** Sample traces from multiple ROIs from astrocytes as in **(E)**. Note that the Y scale is inverted as decreased Flamindo2 fluorescence indicates increasing cAMP. **(G)** Flamindo2 fluorescence with increasing concentrations of adenosine (n = 46-234). **(H)** Concentration-response relationship of cAMP (Flamindo2 fluorescence change) vs. adenosine concentration. Data values were fitted using a function for a sigmoidal curve. **(A)** created using Biorender.

The olfactory bulb is the most rostral part of the mouse brain and receives afferent input from olfactory receptor neurons (ORNs) located in the olfactory epithelium in the nasal cavity (**Figure 1A**) (32). Axons of ORNs terminate in the glomerular layer of the olfactory bulb where they release both ATP and glutamate as neurotransmitters (33) (**Figure 1B**). Astrocytes respond to glutamate and ATP as well as to adenosine derived from ATP with Ca^2+^ signaling, however, whether glutamate, ATP or its degradation product adenosine stimulates cAMP signaling in olfactory bulb astrocytes has not been shown before.

To investigate cAMP signaling and visualize cAMP dynamics in astrocytes of the olfactory bulb, we used the genetically encoded cAMP indicator Flamindo2. We expressed Flamindo2 in astrocytes by injection of AAV carrying the Flamindo2 gene (**Figure 1C**). Flamindo2 expression was under control of the gfaABC1D promoter to assure astrocyte-specific expression (34). We verified specific expression in astrocytes by co-labeling of Flamindo2 and astrocytes using anti-GFP and combined anti-GFAP/S100B immunostaining, respectively. Approximately 50% of astrocytes in the glomerular layer expressed Flamindo2 (**Figure 1D**).

**Figure 2:**
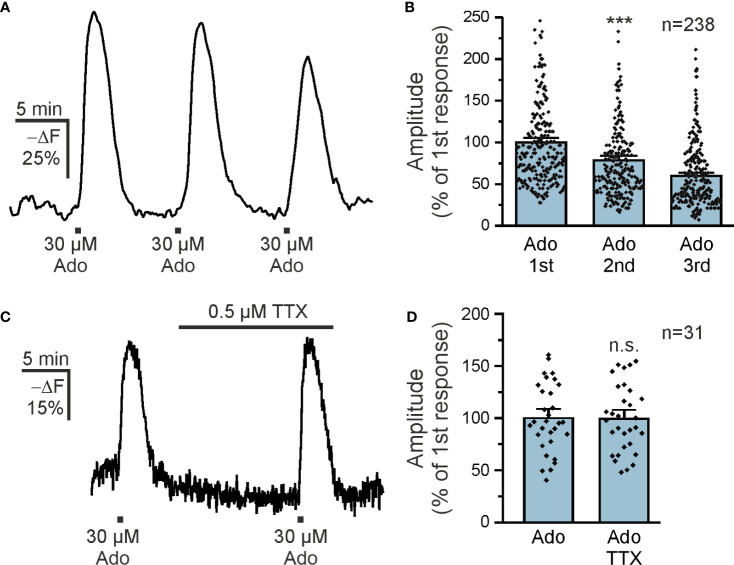
Adenosine directly increases astrocytic cAMP. **(A)** Repetitive applications of 30 µM adenosine repeatedly increased astrocytic cAMP (n = 238). **(B)** Quantification of experiments as in **(A)** indicates a small but significant decrease in the successive responses to adenosine. ***p < 0.001, Kruskal-Wallis followed by Dunn’s multiple comparisons. **(C)** Adenosine-induced cAMP responses in astrocytes in the absence and presence of tetrodotoxin (TTX) to inhibit neuronal spiking (n = 31). **(D)** Quantification of experiments as in **(C)**. n.s., not significant, Mann-Whitney-U test.

Purinergic signaling is strongly involved in a plethora of neurological diseases (35) and we focused on the contribution of purinoceptors in cAMP signaling in olfactory bulb astrocytes. Adenosine activates both G_S_-coupled receptors that stimulate adenylyl cyclases and hence increase the intracellular cAMP concentration and G_i_-coupled receptors that inhibit adenylyl cyclases and thereby decrease the cAMP concentration (36). We applied adenosine to acute brain slices and detected a decrease in Flamindo2 fluorescence in glomerular astrocytes, indicative for an increase in cAMP concentration (**Figure 1E**). To better reflect the changes in cAMP concentration, we inverted the Y axis (-ΔF) (**Figure 1F**). We applied different concentrations of adenosine between 3 and 300 µM and plotted the results in a dose-response chart (**Figures 1G, H**). The results show an EC_50_ value of 12.8 µM, as extrapolated from the sigmoidal fit, and that at 300 µM adenosine the response is close to saturation. For the following experiments, we used 30 µM adenosine, which evokes large non-saturating responses. Repetitive application of adenosine (10 min interval) resulted in cAMP transients with a small yet significant decrease in amplitude (**Figure 2A**). The first application of adenosine evoked a cAMP response with a mean amplitude of 42.5 ± 1.6% -ΔF (n = 238). The amplitude of the responses induced by the second and third applications decreased to 75.8% (p < 0.001) and 59.6% (p < 0.001) compared to the first application (**Figure 2B**). Inhibition of voltage-gated Na^+^ channels with tetrodotoxin (TTX) and thus suppression of neuronal activity had no effect on the adenosine-induced cAMP signals, indicating that the responses in astrocytes did not depend on neuronal neurotransmitter release but were directly evoked in astrocytes (**Figures 2C, D**).

### 3.2 A_2A_ adenosine receptors mediate cAMP signals in astrocytes

**Figure 3:**
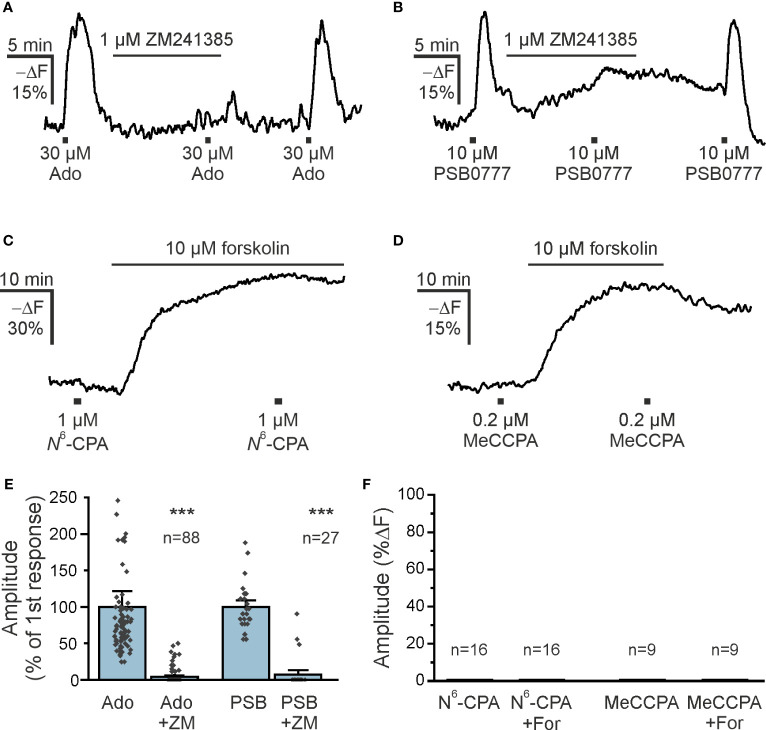
A_2A_ adenosine receptors increase astrocytic cAMP **(A)** The adenosine A_2A_ receptor antagonist ZM241385 inhibits adenosine-induced cAMP increases in astrocytes (n = 88). **(B)** Increases in astrocytic cAMP in response to the specific A_2A_ receptor agonist PSB0777 are inhibited by ZM241385 (n = 27). **(C, D)** The adenosine A_1_ receptor agonists N^6^-CPA (n = 16) and MeCCPA (n = 9) did not affect astrocytic cAMP either alone, or after stimulation of adenylyl cyclases by forskolin. **(E)** Quantification of the effect of ZM241385 (ZM) on cAMP responses evoked by adenosine (Ado) and PSB0777 (PSB). ***p < 0.001, Mann-Whitney-U test. **(F)** Quantification of experiments as in **(C, D)**. N^6^-CPA and MeCCPA had no effect on the cAMP concentration. For, forskolin.

Both A_1_ and A_2A_ receptors are the main adenosine receptors expressed in the olfactory bulb and modulating neuronal performance (31, 37, 38). Olfactory bulb astrocytes express A_2A_ receptors that mediate an increase in the cytosolic Ca^2+^ concentration (39). The canonical pathway downstream of A_2A_ receptor stimulation is activation of adenylyl cyclase resulting in an increase in the cAMP concentration. Therefore, we first tested the effect of the A_2A_ receptor antagonist ZM241385 on adenosine-mediated cAMP increases in olfactory bulb astrocytes in acute brain slices. ZM241385 entirely blocked adenosine-evoked cAMP transients in the majority of astrocytes, indicating that A_2A_ receptors are the main drivers of adenosine-induced cAMP signals (**Figures 3A, E**). This was confirmed by the agonistic effect of PSB0777, a highly specific A_2A_ receptor agonist (40) (**Figures 3B, E**). The PSB0777-evoked increase in cAMP was blocked by ZM241385. It is interesting that in some astrocytes the inhibition by ZM241385 was incomplete, which might be due to local concentration gradients and being outcompeted by the agonist, or might indicate that some astrocytes express an additional adenylyl cyclase-activating adenosine receptor such as A_2B_ (**Figure 3E**). Another widely expressed adenosine receptor found in astrocytes is the A_1_ receptor, that is linked to G_i_ and hence triggers a decrease in cAMP concentration by inhibiting adenylyl cyclases (41). The A_1_ receptor agonist N^6^-CPA, however, neither decreased the cAMP concentration in olfactory bulb astrocytes at resting conditions nor after increasing the cAMP concentration by the adenylyl cyclase activator forskolin (**Figures 3C, F**). Since N^6^-CPA possesses only weak adenosine receptor subtype selectivity, we additionally applied the highly selective and effective A_1_ receptor agonist MeCCPA (42). MeCCPA also failed to induce a decrease in cAMP concentration (**Figure 3D**). Thus, in the majority of astrocytes adenosine elicits cAMP responses by stimulating A_2A_ receptors, while A_1_ receptors are not involved.

### 3.3 Adenosine derived from synaptically released ATP drives cAMP signaling in astrocytes

**Figure 4:**
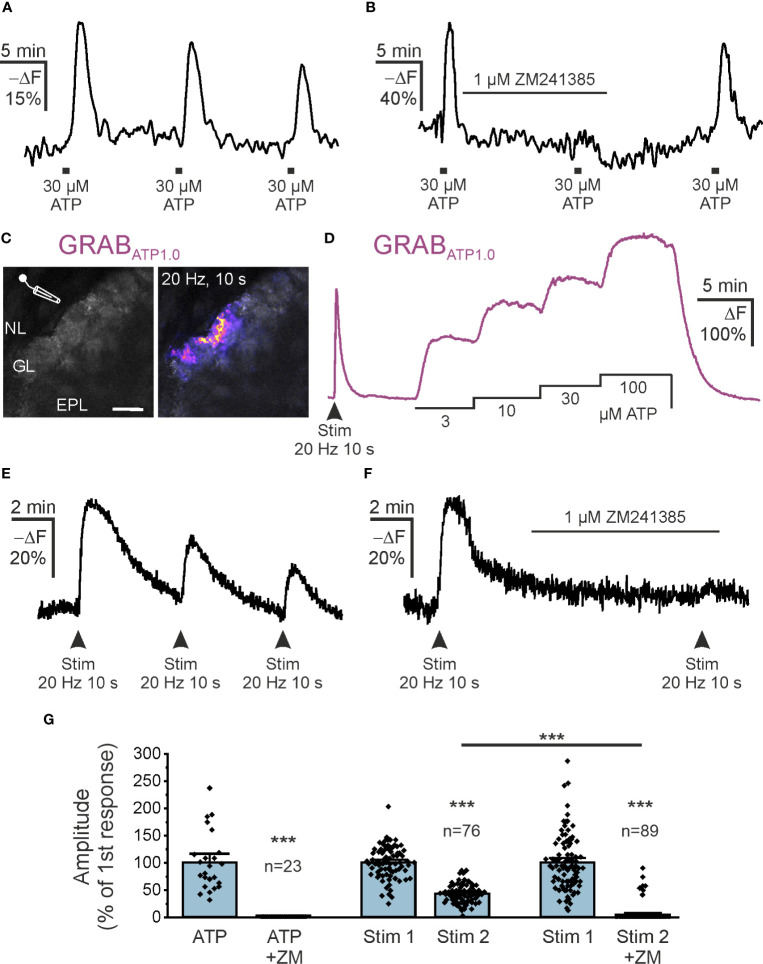
Synaptically released ATP increases astrocytic cAMP via adenosine A_2A_ receptors. **(A)** Flamindo2-indicated astrocytic cAMP responses to bath application of ATP (n = 76). **(B)** The adenosine A_2A_ receptor antagonist ZM241385 inhibits cAMP responses to exogenous ATP (n = 23). **(C)** Expression of the extracellular ATP sensor GRAB_ATP1.0_ (GRAB_ATP_) in olfactory bulb astrocytes and position of electrode used to stimulate neurons. NL, nerve layer; GL, glomerular layer; EPL, external plexiform layer. Scale bar 50 µM. The right panel highlights the GRAB_ATP1.0_ response to electrical stimulation of ORN axons in the NL (20 Hz for 10 s) (n = 10 slices from 4 animals). **(D)** Exemplary responses of GRAB_ATP_ to stimulation of ORN axons and dose-response relationship. Note that neuronal stimulation increases GRAB_ATP_ fluorescence corresponding to more than 10 µM ATP. **(E)** Flamindo2 fluorescence changes demonstrate increased astrocytic cAMP in response to repeated neuronal stimulation. **(F)** ZM241385 inhibits stimulation-induced increases in astrocytic cAMP (n = 89). **(G)** Quantification of experiments as in B, E and F normalized to the first responses to neuronal stimulation. Both responses by bath application of ATP and by neuronal stimulation were inhibited by ZM241385 (ZM). ***p < 0.001, Mann-Whitney-U test.

The main source of extracellular adenosine in the CNS is synaptically released ATP that is degraded by ectonucleotidases, although alternative release mechanisms have also been proposed (43–45). The olfactory bulb receives input from axons of ORNs that are synaptically connected to mitral and tufted cells as well as to some interneurons (32). In the developing olfactory bulb, we have shown that release of ATP from ORN axons and subsequent degradation to adenosine leads to A_2A_ receptor activation and Ca^2+^ signaling in astrocytes of the glomerular layer (39). Therefore, we were interested in whether adenosine could be generated from ATP released from ORN axons and stimulate cAMP signaling in astrocytes in adult mice. We first tested the effect of bath-applied ATP on cAMP in astrocytes of acute brain slices of the olfactory bulb. Application of 30 µM ATP resulted in an increase in intracellular cAMP in astrocytes of the glomerular layer (**Figure 4A**). The mean amplitude of the ATP-evoked cAMP rise was 23.5 ± 1.2% -ΔF (n = 81), which decreased slightly upon repetitive ATP application. The second and third application of ATP resulted in cAMP signals with 79% and 73% (n = 57), respectively, when compared to the first application. The ATP-induced cAMP transients were entirely inhibited by ZM241385 (n = 23), indicating that ATP-evoked cAMP signals were mediated by adenosine acting on A_2A_ adenosine receptors (**Figure 4B**). We used the genetically encoded extracellular ATP sensor GRAB_ATP1.0_, introduced into astrocytes by AAV injection, to visualize changes in extracellular ATP (30). We electrically stimulated ORN axons in the nerve layer of the olfactory bulb and could detect an increase in extracellular ATP in the glomerular layer (**Figure 4C**). We calibrated the GRAB_ATP1.0_ signal by applying ATP at different concentrations via the perfusion system. When compared to the calibration values, the peak of ATP increases evoked by electrical stimulation of axons amounted to values between 3 and 10 µM (**Figure 4D**). On average, the extracellular ATP concentration reached 3.6 µM during axon stimulation (10 slices from 4 animals). We next assessed whether ATP released from ORN axons is able to increase cAMP in astrocytes of the glomerular layer. Electrical stimulation of ORN axons resulted in an increase in astrocytic cAMP of 25.2 ± 1.6% -ΔF (n = 76) (**Figure 4E**). The cAMP response evoked by a second stimulation was significantly smaller and amounted to 43.3 ± 1.8% compared to the first application (n = 76; p < 0.001). When the second stimulation was applied in the presence of ZM241385, no cAMP transients could be measured in the majority of astrocytes (82 out of 89 cells) (**Figure 4F**), while in 7 cells ZM241385 had no effect. In summary, our results show that adenosine produced from bath-applied and synaptically released ATP results in ZM241385-sensitive increases of intracellular cAMP in olfactory bulb astrocytes (**Figure 4G**).

### 3.4 Adenosine-evoked cAMP transients are not altered in experimental autoimmune encephalomyelitis

**Figure 5:**
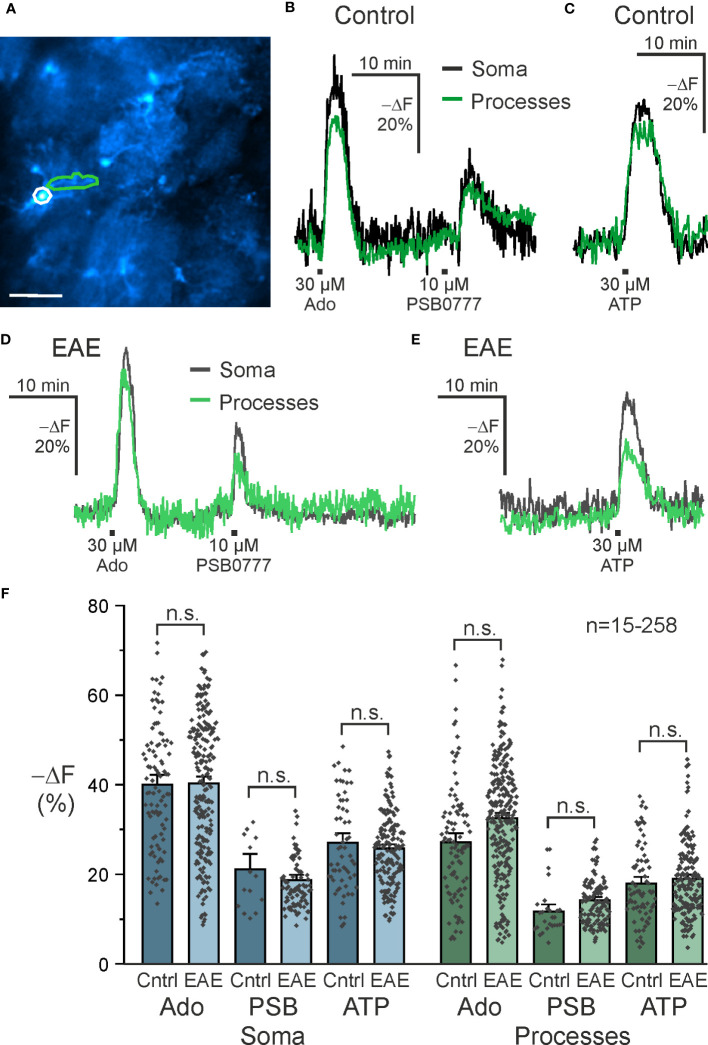
A_2A_-mediated increases in astrocytic cAMP are unaffected in EAE. **(A)** Astrocytes expressing the cAMP indicator Flamindo2 with example soma (white) and process (green) regions of interest indicated. Scale bar 50 µM. **(B)** Exemplary responses to adenosine (Ado) (soma: n = 97; processes: n = 95), the specific A_2A_ receptor agonist PSB0777 (soma: n = 15; processes: n = 27), and **(C)** ATP (soma: n = 61; processes: n = 75) in control mice and **(D, E)** experimental autoimmune encephalomyelitis (EAE) mice (Ado, soma: n = 221; processes: n = 258; PSB0777, soma: n = 88; processes: n = 125; ATP, soma: n = 173; processes: n = 189). **(F)** Quantification of experiments as in B-E. Mean ± SEM and individual peak responses are shown. n.s., not significant, Kruskal-Wallis followed by Dunn’s multiple comparisons.

The role of A_2A_ receptors in astrocytes in neurodegenerative diseases such as Alzheimer’s and Parkinson’s disease is well described (46–48). Less is known about astrocytic A_2A_ receptors in neuroinflammation and we used EAE mice as a model to study purinergic signaling in neuroinflammation. Purinergic signaling in astrocytes is able to mediate both pro- and anti-inflammatory responses and we were interested in whether purinergic cAMP signaling in astrocytes is altered in olfactory bulb astrocytes in the acute phase of EAE. We applied adenosine, the A_2A_-specific agonist PSB0777 and ATP and compared cAMP responses in astrocytes in acute slices of healthy control mice with those of mice suffering from EAE at the peak of the disease, i.e. day 14 to 16 post immunization. To gain additional information of the cAMP responses, we analyzed cAMP signals in somata and cell processes of olfactory bulb astrocytes separately (**Figure 5A**). The amplitude of cAMP rises in somata was significantly larger compared to the cell processes using either adenosine or ATP (**Supplementary Table 1**; **Figures 5B, C**). Similar results were found in brain slices from animals with EAE (**Figures 5D, E**; **Supplementary Table 1**). We compared the amplitudes of the cAMP responses between healthy mice and mice with EAE but did not detect any significant differences, regardless whether we tested the different agonists or whether either values from somata or cell processes were compared (**Figure 5F**). Hence, at least in olfactory bulb astrocytes, EAE appears not to have a major impact on purinergic cAMP signaling.

## 4 Discussion

In the present study, we investigated the contribution of purinergic signaling to cAMP dynamics in olfactory bulb astrocytes. Our results show that ATP released from afferent axons is degraded to adenosine that stimulates A_2A_ adenosine receptors in astrocytes of the glomerular layer. A_2A_ receptor activation results in an increase in intracellular cAMP. This cAMP response was not altered in amplitude in mice suffering from EAE.

### 4.1 Olfactory bulb astrocytes express A_2A_ receptors linked to adenylyl cyclase stimulation

Bath application of adenosine reliably evoked cAMP transients in olfactory astrocytes that were not affected when neuronal activity was blocked using TTX, indicating that adenosine directly stimulated astrocytes. Adenosine-induced cAMP increases were entirely blocked by ZM241385, an A_2A_-preferring adenosine receptor antagonist (49), in the majority of astrocytes. However, at the concentration used (1 µM), ZM241385 could also inhibit other subtypes of adenosine receptors. Therefore, we applied the selective A_2A_ agonist PSB0777 to verify the presence of A_2A_ receptors in olfactory bulb astrocytes (40). PSB0777 also evoked increases in cAMP, indicating the presence of A_2A_ in olfactory bulb astrocytes. In addition, in some astrocytes, ZM241385 reduced adenosine-evoked cAMP responses only partially, which could suggest the contribution of another adenosine receptor such as the A_2B_ receptor. However, this is unlikely as cAMP responses evoked by the A_2A_-selective antagonist PSB0777 were also only partially reduced by ZM241385 in some cells favoring an incomplete inhibition of the A_2A_ receptor. A_1_ receptors are linked to inhibition of adenylyl cyclase and hence lead to a reduction of the cAMP concentration, thereby counteracting A_2A_ receptors. In our study, we applied two A_1_ receptor agonists, N^6^-CPA and MeCCPA, that did not decrease the cAMP concentration. Even when the resting cAMP concentration was artificially increased by the adenylyl cyclase activator forskolin, stimulation of A_1_ receptors had no effect on the cAMP concentration, indicating that glomerular astrocytes do not express A_1_ receptors linked to adenylyl cyclase inhibition. We cannot exclude the contribution of A_3_ receptors to adenosinergic cAMP dynamics in the astrocytes (50, 51), but the strong effect of ZM241385 on adenosine-evoked cAMP signals suggests that A_2A_ receptors are the main players in purinergic cAMP signaling in these cells. We found an EC_50_ value of 12.8 µM of the adenosinergic cAMP response, which is about twentyfold higher than published EC_50_ values of A_2A_ receptor activation. However, adenosine is rapidly degraded to inosine by extracellular adenosine deaminase or taken up by adenosine transporters (52), hence the adenosine concentration reaching astrocytic A_2A_ receptors may be much lower than the adenosine concentration in the perfusion saline. In summary, our results indicate that glomerular astrocytes express A_2A_ receptors that stimulate adenylyl cyclase and increase the cAMP concentration. Astrocytes in the olfactory bulb have been shown to affect neuronal performance and mediate neurovascular coupling in the olfactory bulb, mostly triggered by Ca^2+^ signaling (53–58). Which role astrocytic cAMP signaling plays in the olfactory bulb neuronal network still needs to be shown.

### 4.2 Synaptic release of ATP in the glomerular layer leads to A_2A_ receptor activation and cAMP signaling in astrocytes

In the developing mouse olfactory bulb, it has been shown that axons of ORNs release ATP that triggers Ca^2+^ signaling in two types of glial cells, namely astrocytes and olfactory ensheathing cells, by activation of P2Y_1_ receptors (39, 59, 60). In addition, in the glomerular layer ATP is degraded to adenosine that stimulates Ca^2+^ signals in developing astrocytes via A_2A_ receptors (39). We now studied the effect of ATP release on astrocytes in olfactory bulbs of adult mice. Using the fluorescent ATP sensor GRAB_ATP_ we were able to visualize extracellular ATP and to prove that also in adult mice, ORNs release ATP at their terminals in the glomerular layer. The peak of the extracellular ATP increase was approximately 10 µM which was in the range of the concentrations of ATP and adenosine used in the present study for bath application. Responses in cAMP evoked by both bath-applied ATP and synaptically released ATP were entirely blocked by ZM241385 in the majority of astrocytes, indicating that synaptically released ATP is degraded to adenosine that stimulates A_2A_ receptors, while other adenosine receptors or P2Y receptors play only a minor role in purinergic cAMP signaling in olfactory bulb astrocytes.

### 4.3 A_2A_-mediated cAMP signaling in olfactory bulb astrocytes is not affected in EAE

In neuroinflammation, astrocytes undergo a severe morphological transformation, hallmarked by proliferation, growth of cell processes and up-regulation of the expression of intermediate filaments such as glial fibrillary acidic protein (GFAP) and vimentin (6, 61–63). In addition, in a neuroinflammatory and neurodegenerative environment, purinergic Ca^2+^ signaling can be enhanced (15, 64, 65). We checked whether also purinergic cAMP signaling might be affected by neuroinflammation in the olfactory bulb. We studied neuroinflammation in EAE, a model for MS, since it has been shown that astrocytes are involved in pathogenesis and progression of the disease in the spinal cord and brain of both MS and EAE (8, 10). For example, inhibition of A_2A_ receptors in EAE ameliorated neurological and behavioral deficits in diseased mice (66, 67). We did not find any differences between A_2A_-mediated cAMP signals in olfactory bulb astrocytes of healthy mice compared to mice suffering from EAE, indicating that adenosinergic cAMP signaling in astrocytes is not up- or down-regulated in EAE in this brain region. A_2A_ receptors on other cell types or pathways downstream of cAMP in olfactory bulb astrocytes are therefore more likely contributing to disease severity in EAE. We can also not exclude an involvement of astrocytic A_2A_-dependent cAMP signaling in other brain regions or in the spinal cord in EAE. Furthermore, other components of the purinergic transmitter system could be affected by EAE and differences in the amount of extracellular adenosine and in the expression levels of ATP-degrading enzymes could also account for an impact on purinergic signaling in EAE but have not been addressed in the present study (68). Several studies have shown an important role of A_2A_ receptors in EAE. Of note, A_2A_ receptors can have both beneficial and exacerbating effects on the severity of EAE. A_2A_ stimulation inhibits lymphocyte proliferation and release of proinflammatory cytokines that promote MS progression, while inhibition of A_2A_ in EAE mice attenuated the progression of EAE and A_2A_-deficient mice developed a more severe acute phase of EAE (66, 67, 69, 70). In other neurological diseases and disease models, an up-regulation of A_2A_ in astrocytes and contribution of A_2A_ to the disease progression has been demonstrated (46–48). In a mouse model of Alzheimer’s disease (AD), up-regulation of A_2A_ receptors in hippocampal astrocytes was shown which can lead to dysregulation of gene expression and connexin43-dependent gliotransmitter release in astrocytes (71–73). In addition, in an animal model of chronic cerebral hypoperfusion inducing neuroinflammation, activation of astrocytic A_2A_ receptors reduced the pro-inflammatory signal transducer and activator of transcription 3 (STAT3)/Chitinase 3-like-1 (YKL-40) pathway, resulting in decreased release of pro-inflammatory cytokines (74). These examples imply that A_2A_ receptors in astrocytes play an important role in neurodegeneration and neuroinflammation, however, the role of astrocytic A_2A_ receptors in MS and EAE needs still to be elucidated. Our results indicate that A_2A_ receptors in olfactory bulb astrocytes significantly contribute to cAMP dynamics but do not provide evidence for dysregulation of A_2A_ receptor expression and function in EAE.

## Data availability statement

The raw data supporting the conclusions of this article will be made available by the authors, without undue reservation.

## Ethics statement

The animal study was approved by Behörde für Justiz und Verbraucherschutz der Freien und Hansestadt Hamburg Lebensmittelsicherheit und Veterinärwesen Billstraße 80a 20539 Hamburg Germany. The study was conducted in accordance with the local legislation and institutional requirements.

## Author contributions

MW: Formal Analysis, Writing – review & editing, Investigation. AK: Formal Analysis, Investigation, Writing – review & editing. AB: Formal Analysis, Investigation, Writing – review & editing, Conceptualization, Supervision. CS: Conceptualization, Formal Analysis, Investigation, Supervision, Writing – review & editing. KS-Y: Formal Analysis, Investigation, Writing – review & editing. JS: Writing – review & editing, Data curation, Formal Analysis. MP: Writing – review & editing, Investigation, Methodology. KS: Supervision, Writing – review & editing. MF: Conceptualization, Funding acquisition, Project administration, Supervision, Writing – review & editing. CG: Conceptualization, Funding acquisition, Project administration, Supervision, Writing – review & editing, Methodology. DH: Conceptualization, Data curation, Funding acquisition, Project administration, Supervision, Writing – review & editing, Investigation. CL: Conceptualization, Data curation, Formal Analysis, Funding acquisition, Methodology, Project administration, Supervision, Visualization, Writing – original draft, Writing – review & editing.
