# Supplementary material for: A2A adenosine receptor-driven cAMP signaling in olfactory bulb astrocytes is unaffected in experimental autoimmune encephalomyelitis
Source: Front Immunol. 2023 Nov 23;14:1273837. doi: 10.3389/fimmu.2023.1273837 (PMC10701430; doi:10.3389/fimmu.2023.1273837)
Supplement: Supplementary file 1 [file Table_1.docx]

Supplementary Material

**A_2A_ adenosine receptor-driven cAMP signaling in olfactory bulb astrocytes is unaffected in experimental autoimmune encephalomyelitis**

Marina Wendlandt^1^, Alina J. Kürten^1^, Antonia Beiersdorfer^1^, Kiana Samad Yazdtchi^1^, Jessica Sauer^1^, M. Carolina Pinto^2^, Kristina Schulz^1^, Daniela Hirnet^1^, Charlotte Schubert^3^, Manuel A. Friese^3^, Christine E. Gee^2^, Christian Lohr^1*^

*** Correspondence:** Corresponding Author: christian.lohr@uni-hamburg.de

# Supplementary Table 1. Differences in amplitude of cAMP transients evoked by adenosine, PSB0777 and ATP between somata and cell processes in control mice and EAE mice, as tested with Mann-Whitney-U test. All mean ± SEM values in % -ΔF.

|  |  | **Soma** | | |  | **Processes** | | |  |
| --- | --- | --- | --- | --- | --- | --- | --- | --- | --- |
| **Control** |  | **Mean** | **SEM** | **N=** |  | **Mean** | **SEM** | **N=** | **p=** |
|  | **Ado** | 40.1 | 1.47 | 97 |  | 27.1 | 1.36 | 95 | 3.714E-9 |
|  | **PSB** | 20.6 | 2.08 | 15 |  | 11.7 | 1.04 | 27 | 2.635E-4 |
|  | **ATP** | 27.1 | 1.41 | 61 |  | 17.9 | 0.98 | 75 | 8.354E-7 |
| **EAE** |  |  |  |  |  |  |  |  |  |
|  | **Ado** | 40.3 | 1.01 | 221 |  | 32.5 | 0.83 | 258 | 1.386E-8 |
|  | **PSB** | 18.6 | 0.77 | 88 |  | 14.2 | 0.52 | 125 | 2.294E-7 |
|  | **ATP** | 25.8 | 0.65 | 173 |  | 19.0 | 0.61 | 189 | 4.690E-14 |
